# GERIATRIC ASSESSMENT BEFORE HEMATOPOIETIC STEM CELL TRANSPLANT IDENTIFIES DEFICITS ACROSS ALL AGES

**DOI:** 10.1093/geroni/igz038.1723

**Published:** 2019-11-08

**Authors:** yen p lowder, Kristi Romero, Yi Ren, Amy M Pastva, Miriam C Morey, Harvey J Cohen, Nelson J Chao, Anthony D Sung

**Affiliations:** 1 Duke University Hospital, Durham, North Carolina, United States; 2 Durham Veterans Affairs Health Care system, Durham, North Carolina, United States; 3 Duke University Medical Center, Durham, North Carolina, United States

## Abstract

Allogeneic hematopoietic stem cell transplant (HCT) is a lifesaving procedure; however, it is associated with significant morbidity, and treatment-related mortality ranges from 10-30%. Morbidity and mortality have been associated with poor functional status. The geriatric assessment (GA) may allow identification of deficits pre-HCT, allowing intervention and improvement. While focused on older adults, we hypothesize that GA may also identify deficits in younger patients who may be debilitated by chemotherapy or cancer before HCT. We performed a GA in all adult patients at the time of initial evaluation for HCT (between 10/1/17-1/31/19) and again immediately before HCT. Deficits were identified and patients referred to specialists (physical therapy, neuropsychology, etc.) prior to HCT. Among 83 patients, the median age was 58 years (age range: 19-75), 59 (71%) had ≥1 deficits, including 41 (49%) had ≥2 deficits that required referral. The most common deficit was physical function (45, 54%), followed by cognitive function (29, 35%), nutrition (26, 31%), and mental health (7, 8%). Deficits were common across all age groups: 9/16 (56%) 60 years old. To date, 40 patients have undergone HCT; of the 24 with deficits at initial evaluation, 10 (42%) improved at least one deficit, 5 (21%) were unchanged, and 9 (38%) not evaluated. Physical and nutrition deficits were most responsive to intervention. These results suggest that there is a high degree of impairment prior to HCT among both older and younger patients; however, these deficits are amenable to improvement prior to HCT.

